# Prevalence of aphthous stomatitis in patients with inflammatory bowel disease after the treatment with monoclonal antibodies: a systematic review and meta-analysis

**DOI:** 10.4317/medoral.25528

**Published:** 2022-09-29

**Authors:** Angel Orión Salgado-Peralvo, María Montero-Alonso, Naresh Kewalramani, Mario Pérez-Sayáns-García, María Victoria Mateos-Moreno, María Rosario Garcillán-Izquierdo

**Affiliations:** 1 ORCID: 0000-0002-6534-2816. DDS, MSc, MSc, PhD. Department of Dental Clinical Specialties, Faculty of Dentistry, Complutense University of Madrid, Spain; 2 ORCID: 0000-0003-0116-3402. PD, MSc, MSc. Pediatric Service, Hospital Álvaro Cunqueiro. Vigo, Pontevedra, Spain; 3 ORCID: 0000-0002-5162-2841. DDS, MSc. Department of Nursery and Stomatology, Faculty of Dentistry, Rey Juan Carlos University. Madrid 28933, Spain; 4 ORCID: 0000-0003-2196-9868. DDS, MSc, PhD. Department of Surgery and Medical-Surgical Specialties, Faculty of Dentistry, University of Santiago de Compostela 15782, Spain; 5 ORCID: 0000-0002-8237-4596. DDS, MSc, PhD. Department of Dental Clinical Specialties, Faculty of Dentistry, Complutense University of Madrid 28040, Spain; 6 ORCID: 0000-0002-2258-6597. DDS, PhD. Department of Dental Clinical Specialties, Faculty of Dentistry, Complutense University of Madrid 28040, Spain

## Abstract

**Background:**

Currently, the most frequently employed therapies in the treatment of inflammatory bowel diseases (IBD), i.e., Crohn's Disease (CD), Ulcerative Colitis (UC) or unclassified IBD (IBD-U) are monoclonal anti-TNFs and anti-integrin therapies, such as vedolizumab (VDZ). Forty-seven per cent of these patients present extra-intestinal manifestations, the second most prevalent being aphthous stomatitis (AS). The present study aims to investigate which of the two therapies is associated with a lower prevalence of AS after treatment.

**Material and Methods:**

An electronic search of the MEDLINE (via PubMed), Web of Science, SCOPUS, LILACS and OpenGrey databases was carried out. The criteria used were those described by the PRISMA Statement. The search was not temporarily restricted and was updated to January 2022. The quality assessment was analyzed using the JBI Prevalence Critical Appraisal Tool.

**Results:**

After searching, 7 studies were included that met the established criteria. Of these, 6 analysed the prevalence of AS in CD patients and 4 in UC. A total of 1,744 patients were analysed (CD=1,477 patients; 84.69%; UC=267; 15.31%). The greatest reduction in AS prevalence was observed after anti-TNF therapy. The effect of these therapies on the prevalence of AS in patients with IBD-U could not be determined.

**Conclusions:**

Both biologic therapies achieve a reduction in the prevalence of AS in IBD patients (CD and UC). However, the best results were obtained in patients treated with anti-TNFs, possibly because VDZ is often used in patients who do not respond adequately to previous treatment with anti-TNFs and because of its intestinal specificity.

** Key words:**Inflammatory bowel disease, crohn's disease, ulcerative colitis, undetermined, systemic extraintestinal manifestations, aphthous stomatitis.

## Introduction

Inflammatory bowel diseases (IBD) are chronic, disabling and progressive diseases that primarily affect the gastrointestinal tract (1). They have been classified into three subtypes: Crohn's disease (CD), ulcerative colitis (UC) and a third, IBD-unclassified or undetermined (IBD-U). Globally, its prevalence has increased substantially in many regions from 1990 to 2017. Specifically, there were 6.8 million cases worldwide in 2017, with the highest rates in the USA, with a prevalence rate of 464.5 per 100,000 individuals, followed by the UK, with 449.6 per 100,000 individuals (2).

Furthermore, around 47% of these patients will develop systemic extra-intestinal manifestations (EIMs) (3), which harm the patient's quality of life and influence their treatment (4). Typical EIMs include dermatological (erythema nodosum, pyoderma gangrenosum), ocular (uveitis), hepatopancreatic biliary (primary sclerosing, cholangitis), musculoskeletal system (peripheral arthritis, axial arthropathy), haematological, and even at the level of the oral cavity) (3), such as aphthous stomatitis (AS), mucosal nodularity (cobblestoning), and pyostomatitis vegetans. The most frequent are peripheral arthritis followed by AS, axial arthropathy, uveitis and skin manifestations (5). These manifestations are associated to a greater extent with long-standing IBDs (6), to a greater extent with CD (3), and follow a parallel or separate course from the underlying IBD (7). In particular, AS often evolves alongside IBD, so that its course will follow that of the disease itself (8).

Although the pathogenesis of IBD is not fully understood, immunophenotyping of the inflammatory infiltrate of the intestinal mucosa revealed high cellular activation, especially of CD4+ T cells (9). As a result, these cells synthesize a large number of cytokines, observing a polarity against Th1 type with tumour necrosis factor-alpha (TNFα) playing a key role in this inflammation process. Therefore, TNFα production in the intestinal mucosa is increased in these patients, which correlates with the severity of the disease (10,11). Therefore, for approximately two decades, monoclonal anti-TNF antibodies such as adalimumab, infliximab, certolizumab and golimumab have been used (8), allowing control of systemic inflammation (12,13), however, up to 45% of patients fail to respond during treatment (14) and around two-thirds are unable to achieve or maintain disease remission within one year of starting these therapies (15,16). In these cases, treatment with a second anti-TNF substantially decreases treatment response (17). Therefore, other types of monoclonal antibodies, such as anti-integrin, were developed. This group includes vedolizumab (VDZ), approved in 2014 by the Food and Drug Administration (FDA) (8), which acts by inhibiting gut-targeted leukocyte migration (18). Specifically, it selectively inhibits the adhesion of leukocyte integrin α4ß7 to its main ligand, the mucosal addressin cell adhesion molecule 1 (MAdCAM-1) since, in these patients, this molecule is highly upregulated in high endothelial venules of inflammatory sites and promotes an increased capacity to bind leukocytes (19). To date, it is the only drug for the treatment of IBD that acts selectively in the intestinal tract, so its efficacy in the prevention of EIMs may be limited (18,20,21).

The present systematic review and meta-analysis aimed to compare the prevalence of AS after treatment with anti-TNFs versus anti-integrin monoclonal antibodies to determine which therapy is more effective in reducing, the prevalence of AS.

## Material and Methods

- Protocol and registration

The present systematic review with meta-analysis is reported according to the Preferred Reporting Items for Systematic Reviews and Meta-Analysis (PRISMA) Statement (22), and its protocol was registered on PROSPERO (Registration number: CRD42022308040).

- Initial hypothesis

Given the specificity of anti-integrin monoclonal antibodies (VDZ) at the level of the intestinal tract and their use in cases where the patient has not responded adequately to the treatment of previous IBD with anti-TNF, it is hypothesised that the prevalence of AS following treatment with anti-integrin will be higher than in those treated with anti-TNF.

- Focused question

The study aimed to answer the following PICO (*P*=patient/problem/population; I=intervention; C=comparison; O=outcome) questions (Table 1): 

Q1: In patients with CD (P) treated with anti-TNF monoclonal antibodies (I) compared to anti-integrin monoclonal antibodies (C), which therapy is associated with a lower prevalence of AS (O)?

Q2: In patients with UC (P) treated with anti-TNF monoclonal antibodies (I) compared to anti-integrin monoclonal antibodies (C), which therapy is associated with a lower prevalence of AS (O)?

Q3: In patients with IBD-U (P) treated with anti-TNF monoclonal antibodies (I) compared to anti-integrin monoclonal antibodies (C), which therapy is associated with a lower prevalence of AS (O)?

- Clinical relevance

Although IBD is a well-studied disease for which evidence has increased exponentially in recent decades, the influence of biological therapies based on monoclonal antibodies on oral manifestations such as AS has not been specifically analysed.

- Eligibility criteria

Inclusion criteria: (a) studies conducted in humans; (b) articles published in English and Spanish; (c) clinical trials; (d) controlled clinical trials; (e) randomized clinical trials; (f) multicentre studies; (g) observational studies; (h) clinical studies; (i) clinical trial, Phase I, II, III or IV; (j) comparative studies; and (k) studies with a minimum of 10 participants per type of IBD.

Exclusion criteria: (a) experimental laboratory studies; (b) animal studies; (c) meta-analysis; (d) systematic reviews; (e) studies that do not directly or indirectly assess the influence of these drugs on AS; (f) studies that do not specify the sample size according to the type of IBD or the type of biological drug used; (g) duplicate articles; (h) books or chapters of books; (i) letters to the Editor; (j) commentaries; (k) case reports; and (l) narrative literature reviews.

**Table 1:**
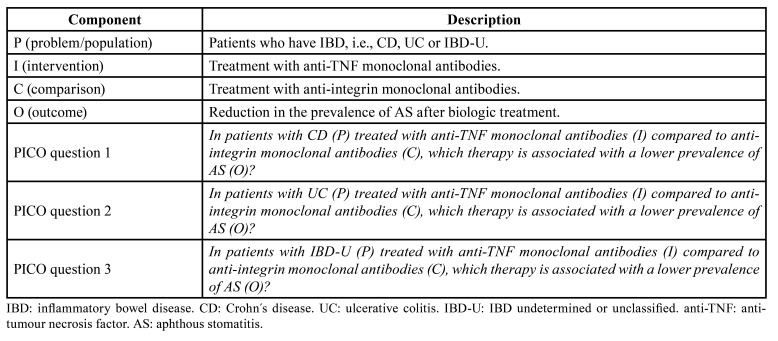
Breakdown of the “PICO” question.

- Information sources and search strategy

A comprehensive search of the literature was conducted in the following databases: Medline (via PubMed), Web of Science, Scopus and LILACS. A search for unpublished studies (grey literature) was conducted on the OpenGrey database. The search was performed by two independent researchers (A.-O.S.-P. and M.M.-A.). The search was not temporarily restricted and was updated to 14th January 2022.

MeSH (Medical Subject Headings) terms, keywords and other free terms were used with Boolean operators (OR, AND) to combine searches: ("Inflammatory Bowel Diseases"[MeSH Terms] OR "colitis, ulcerative"[MeSH Terms] OR "Crohn Disease"[MeSH Terms] OR "inflammatory bowel disease"[Title/Abstract] OR "crohn´s disease"[Title/Abstract] OR "ulceratuve colitis"[Title/Abstract] OR "undetermined"[Title/Abstract]) AND ("Adalimumab"[Mesh] OR "Infliximab"[Mesh] OR "Antibodies, Monoclonal"[Mesh] OR "vedolizumab" [Supplementary Concept] OR vedolizumab[Title/Abstract] OR anti-tnf therapy[Title/Abstract] OR anti-tnf treatment[Title/Abstract] OR anti-tnf[Title/Abstract] OR biological drugs[Title/Abstract] OR monoclonal antibody therapy[Title/Abstract] OR alpha4beta7 integrin antibody[Title/Abstract] OR integrin antibody[Title/Abstract]) AND ("Stomatitis, Aphthous"[Mesh] OR recurrent aphthous stomatitis OR aphthae OR stomatitis OR aphthous stomatitis). The same keywords were used for all search platforms and followed the syntax rules for each database.

- Study records

Two researchers (A.-O.S.-P. and M.M.-A.) independently compared the results to ensure completeness and removed duplicates. Then, the full title and abstracts of the remaining papers were screened individually. Finally, full-text articles to be included in this systematic review were selected according to the criteria described above. Disagreements over eligible studies to be included were discussed with a third reviewer (N.K.) and a consensus was reached. The reference list of the included studies was also reviewed for possible inclusion. Agreement between reviewers was measured with the Kappa coefficient. The results were also expressed as the concordance between reviewers (%).

- Data extraction

Data from included studies were extracted by two review authors (A.-O.S.-P and M.M.-A.) independently and using predefined data extraction forms. Any disagreement was discussed by the two review authors and a third review author (M.-V.M.-M.) was consulted when a resolution was not possible. If necessary, study authors were contacted for clarification or missing information. For each study the following data were recorded: authors and year, country, centre, study type, biological therapy analysed, other drugs with which patients have been treated in the past or currently that may have improved the prevalence of AS, target population, disease severity and/or activity, age of patients included and, for each therapy, sample size, mean duration of therapy and prevalence of AS pre- and post-biological therapy.

- Quality assessment and risk of bias

Two independent reviewers (A.-O.S.-P. and M.M.-A.) evaluated the methodological quality of eligible studies following the Joanna Briggs Institute (JBI) Prevalence Critical Appraisal Tool (23), which incorporates 10 domains. The studies were classified as low-quality assessment studies (0–5 domains), or as high-quality assessment studies (6–10 domains).

- Statistical analysis

A descriptive analysis of the data was performed using frequencies and percentages for categorical variables and mean or median, standard deviation (SD) or ranges (depending on normality fit) for continuous variables. A random-effects model meta-analysis was used to estimate the pooled odds ratio (OR) for the prevalence of AS with the use of anti-TNFs and anti-integrin monoclonal antibodies in each IBD, at 95% confidence intervals (CI). We examined the heterogeneity across studies using the I2 statistic. The results are expressed as Z-score assuming a statistically significant difference with a *p-value* < 0.05.

Statistical treatment of the data was performed with XLSTAT 2018.1 (Microsoft, WA, USA) and a meta-analysis was run with Review Manager (RevMan 5.4., The Cochrane Collaboration, Copenhagen, DK).

## Results

- Study selection

**Figure 1:**
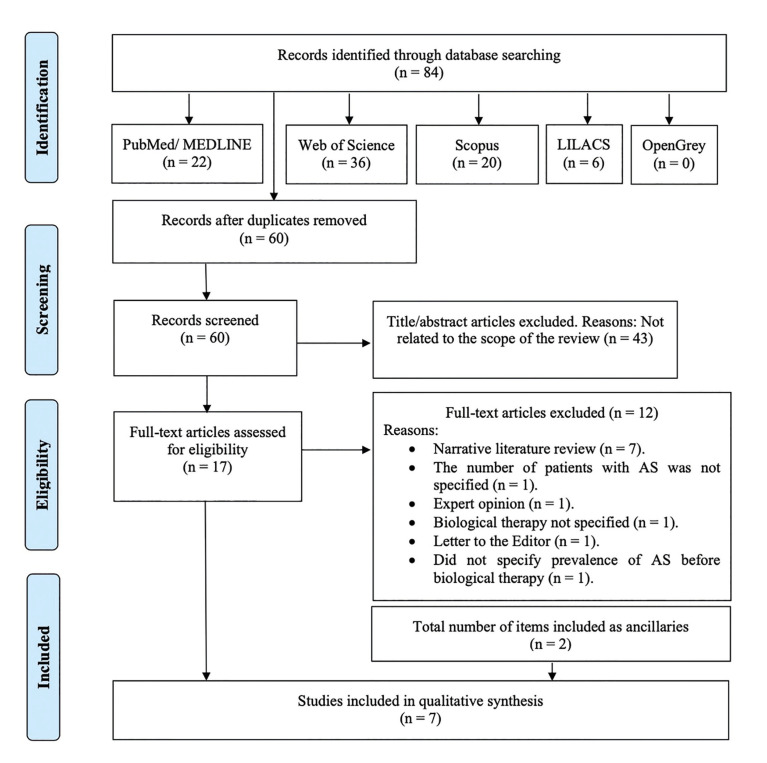
PRISMA flow diagram of the search processes and results.

The search strategy resulted in 84 results, of which 60 remained after removing the duplicates. Then, two independent researchers (A.-O.S.-P. and M.M.-A.) reviewed all the titles and abstracts and excluded 43 that were outside the scope of this review. Thus, we obtained 17 potential references. After reading the full text of those 17 papers, 12 were discarded for being narrative literature reviews (n=7) (24-30), for not specifying the number of patients with AS (n=1) (31), for only indicating the prevalence of AS following biologic treatment (n = 1) (8), for being an expert opinion (n=1) (32), for not specifying the biologic therapy used (n=1) (33), and for being a letter to the Editor (n=1) (34). When analysing the references of the reviewed articles, two articles were included as ancillaries (35,36). Therefore, 7 studies were included in our systematic review (5,19,21,35-38) (Fig. 1).

There was a 97.00% concordance between the two authors (A.-O.S.-P. and M.M.-A.) with a Kappa coefficient of 0.922 (SE 0.054, 95%CI [0.815, 1.028]) for titles and abstracts, and a 100.00% concordance with Kappa coefficient of 1.00 (SE 0.00, 95%CI [1.000, 1.000]) for full-text articles, respectively.

- Characteristics of the included studies

The 7 included studies were published between 2005 and 2021. In terms of publication type and design, the different studies were very diverse. Five of them were cohort studies – one retrospective (37) and four prospective (5,19,21,38) –, one a phase III, multicentre randomised, placebo-controlled, double-blind trial (36) and the remaining study a phase IIIb, multicentre clinical trial (35). Only two studies looked at patients with all three types of IBD (CD, UC and IBD-U) (5,37), one included patients with CD and UC (19), three included only patients with CD (35,36,38) and one included patients with UC (21). Overall, they analysed a total of 1,754 patients, of whom 10 were diagnosed with IBD-U (0.57%). Given the low sample size of the two studies that analysed these patients (<10 participants) and the fact that they only employed anti-TNFs, they were not considered. Therefore, of the remaining 1,744 patients, 1,477 had CD (84.69%), and 267 UC (15.31%). Regardless of the type of IBD, the most studied drugs were anti-TNFs, as they were used in 70.13% of patients (n=1,223) while VDZ was used in 29.87% (n=521).

Disease activity and/or severity were assessed by 5 of the 7 studies (19,21,35,36,38). The way it was determined varied widely. In CD patients, some authors used the Crohn's Disease Activity Index (CDAI) (36), which is a scale measuring 8 components (range, 0 to approximately 600; with higher scores indicating greater disease activity), the Swollen Joint Count (SJC) (38) and the Harvey-Bradshaw Index (HBI) (19).

In UC, on the other hand, the Total Mayo Score (TMS) (range 0–12, with higher scores indicating a more active disease) (21) or Partial (PMS) (TMS without the endoscopic component; range 0–9) (19,21) were used. Primarily patients with moderate to severe IBD were included (19,21,35,38). Two studies did not specify this (5,37).

- Overall prevalence of AS

The overall prevalence of AS in patients with IBD before biologic treatment, regardless of therapy, was 7.97% (n=139/1,744) and, after treatment, 4.36% (n=76/1,744). The results are analysed below according to the type of IBD, and the biological therapy used to answer the PICO questions.

- Prevalence of AS in Crohn´s Disease (CD) 

Six studies investigated the efficacy of biological drugs in reducing the prevalence of AS in CD patients (5,19,35-38). A total of 1,477 patients were analysed: 1,438 adults (97.36%) and 39 paediatric patients (2.64%). The age range varied from 12 to 80 years. Regarding disease severity, four studies included patients with moderate to severe CD (19,35,36,38). Specifically, with 200–400 on the CDAI index (36), an SJC of 10.90±5.80 (38) and an HBI >7 (19). Two authors did not specify (5,37).

One study included patients not previously treated with biological drugs (n=22; 1.49%) (38), while another did (n=165; 11.17%) (5). Four studies included both types (n=1,290 patients; 87.34%) (19,35-37), however, it was not possible to know the number of previously exposed patients.

Of the total number of patients, 306 were given VDZ (20.72%) (19,36) and 1,171 were given anti-TNFs (79.28%) (5,35,37,38). The anti-TNFs administered were adalimumab (35), infliximab (38) and, in another study, certolizumab (5). This was not specified by one author (37). More specifically, the effect of VDZ vs. placebo (36) and, in isolation (no control group) of VDZ (n=1) (19) or anti-TNFs (n=4) (5,35,37,38) was compared. Median follow-up periods ranged from 70 to 542 days. Two studies did not specify the follow-up period (37,38). Finally, the overall prevalence of AS in CD patients was 8.12% (n=120/1,477) pre-biological therapy, and 4.60% (n=68/1,477) post-biological therapy (Table 2).

**Table 2:**
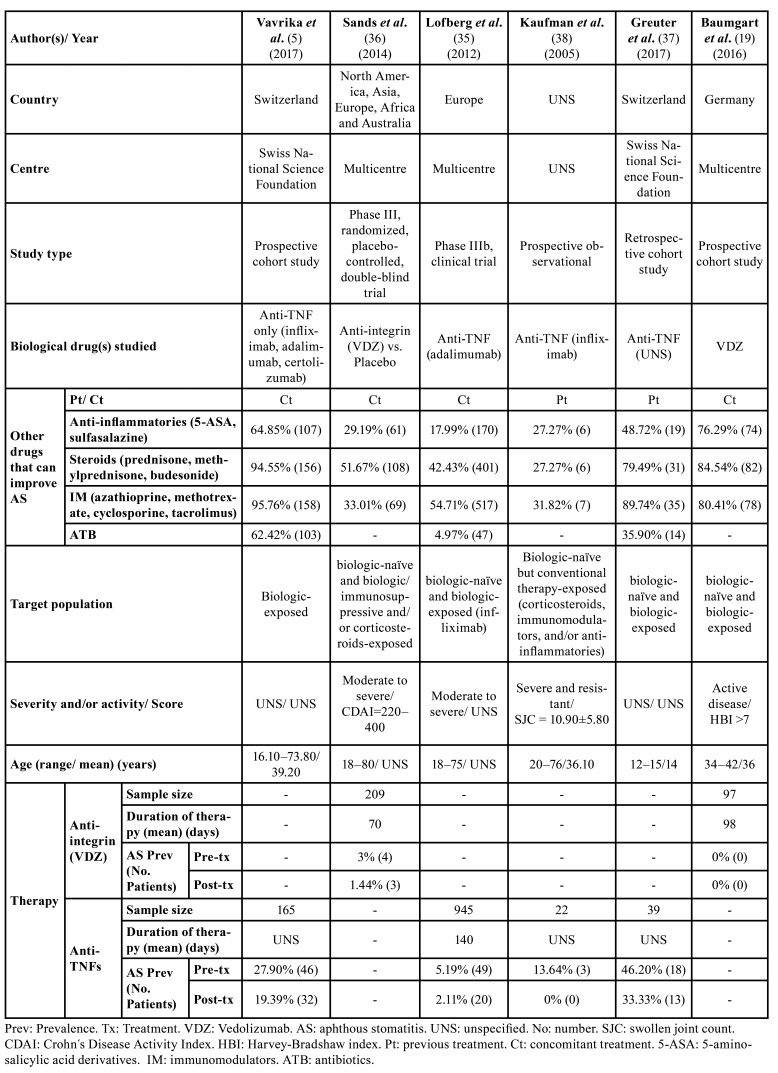
Characteristics of studies included for Crohn´s Disease.

PICO Question 1: After treatment with both biological therapies, there was a decrease in the prevalence of AS. This reduction was greater in those patients treated with anti-TNFs, namely 4.36%, i.e., AS disappeared in 51 patients after treatment, whereas the reduction in patients treated with anti-integrin (VDZ) was 0.33%, with AS disappearing in one patient.

The meta-analysis of the 4 studies (5,35,37,38) evaluating the clinical course of AS before and after anti-TNF treatment gives an overall favourable result in terms of reduction in the number of patients with AS (OR=1.88), with a very wide confidence interval (95%CI 1.24–2.84), with low heterogeneity between studies (I2=18%).

**Figure 2:**
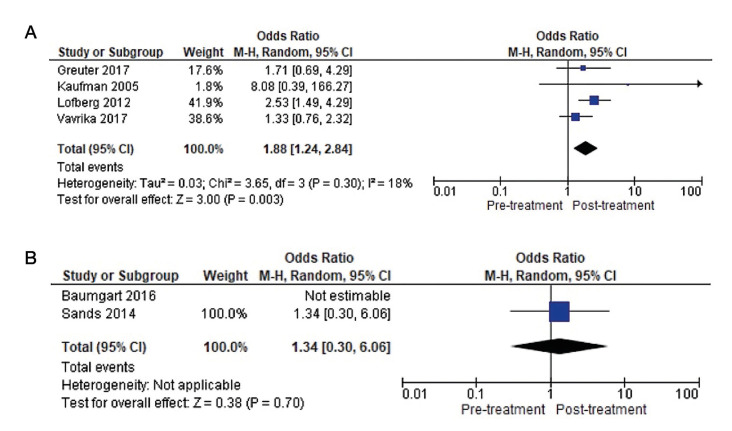
Forest plot of comparison: A) Clinical evolution of AS under anti-TNF therapy in CD patients. B) Clinical evolution of AS under anti-integrin therapy in CD patients.

On the other hand, the meta-analysis of the two studies (19,36) evaluating the effect of anti-integrin (VDZ) treatment showed an overall favourable result in terms of reducing the number of patients with AS (OR=1.34), with a very wide confidence interval (95%CI 0.30–6.06). Heterogeneity between studies is not assessable. Thus, treatment with anti-TNFs shows the greatest reduction in the prevalence of AS compared to anti-integrin (VDZ) therapy (Fig. 2).

- Prevalence of AS in Ulcerative Colitis (UC)

Four studies examined the efficacy of biological drugs on AS in patients with UC (5,19,21,37). A total of 267 patients were analysed: 255 adults (95.51%) and 12 paediatric patients (4.49%). The age range of the included patients was 12 to 73.80 years. Two studies included patients with moderate to severe UC, with an MST of 10 (21) and PMS >4 (19) and 7 (21). Two authors did not provide this data (5,37).

**Table 3:**
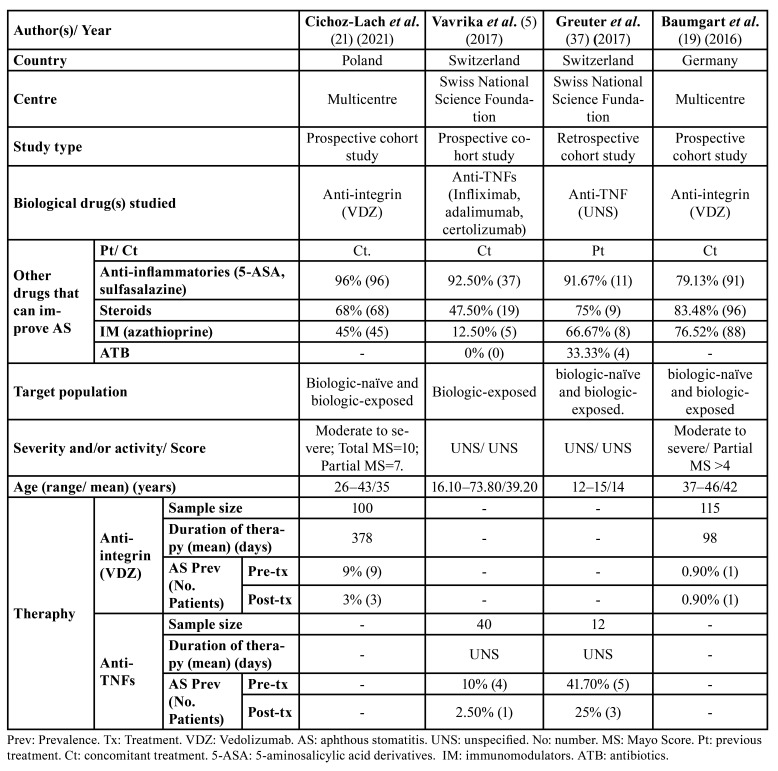
Characteristics of studies included for ulcerative colitis.

Regarding therapy, two studies analysed the efficacy of anti-integrins (VDZ) (19,21) or anti-TNFs (5,37) in isolation. Median follow-up periods ranged from 98 to 502 days. Two studies did not specify (5,37). Only one study included patients previously treated with biological drugs (14.95%, n=40 (5)). Of the total patients included, 215 were treated with VDZ (80.52%) (19,21) and 52 with anti-TNFs (19.48%) (5,37).

Finally, the overall prevalence of AS in UC patients was 7.12% (n=19/267) pre-biological therapy and 3% (n=8/267) post-biological therapy (Table 3).

PICO Question 2: After treatment with both biological therapies, there was a decrease in the prevalence of AS. This reduction was greater in patients treated with anti-TNFs, namely 9.62%, i.e., AS disappeared in 5 patients after treatment, whereas the reduction in patients treated with anti-integrins (VDZ) was 2.79%, disappearing after treatment in 6 patients.

The meta-analysis of the two studies (5,37) evaluating the clinical course of AS before and after treatment with anti-TNFs monoclonal antibodies gives an overall favourable result in terms of reduction in the number of patients with AS (OR=2.79), with a very wide confidence interval (95%CI 0.71–11.03). Heterogeneity between studies is low (I2=0%).

**Figure 3:**
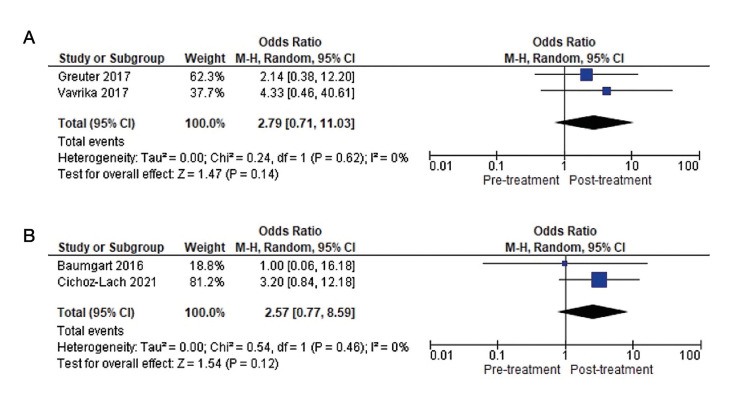
Forest plot of comparison: A) Clinical evolution of AS under anti-TNF therapy in UC patients. B) Clinical evolution of AS under anti-integrin therapy in UC patients.

On the other hand, the meta-analysis of the two studies (19,21) evaluating the effect of anti-integrin (VDZ) treatment showed an overall favourable result in terms of reducing the number of patients with AS (OR=2.57), with a very wide confidence interval (95%CI 0.77–8.59). Heterogeneity between studies is low (I2=0%). Thus, treatment with anti-TNFs shows the greatest reduction in the prevalence of AS compared to anti-integrin (VDZ) therapy (Fig. 3).

- Prevalence of AS in Unclassified or Undetermined Inflammatory Bowel Disease (IBD-U) and PICO Question 3

Two studies included patients with IBD-U (5,37), including a total of 10 patients (0.57%). Given the low sample size per study (<10 participants) and the fact that only anti-TNF monoclonal antibodies were used, these patients were not considered. For this reason, it was not possible to answer the third PICO question.

- Quality assessment and risk of bias

Using the predetermined 10 domains for the methodological quality assessment according to JBI Prevalence Critical Appraisal Tool (23), we determined that 6 of the 7 studies were found to be of high quality (5,19,21,35-37), while the remaining one, was a low-quality (38). Table 4 shows a more detailed description of the articles included.

To assess the presence of a possible publication bias, funnel plots were performed for each of the IBDs evaluated (CD and UC), showing the existence of this bias in a probabilistic manner, due to the asymmetry found in them.

**Table 4:**
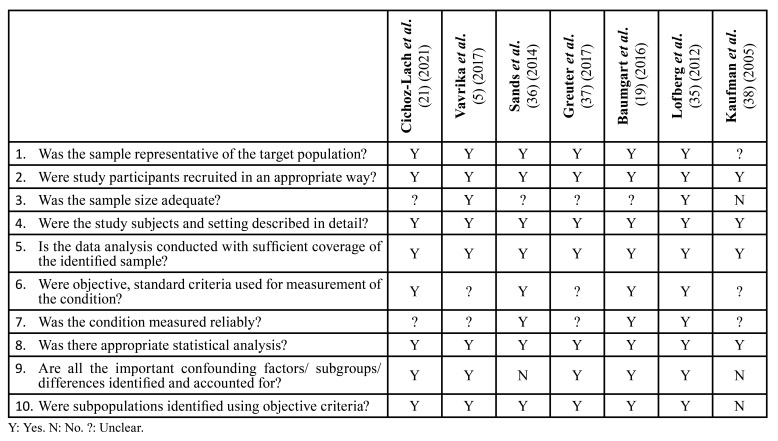
JBI Critical Appraisal Checklist for studies reporting prevalence data.

**Figure 4:**
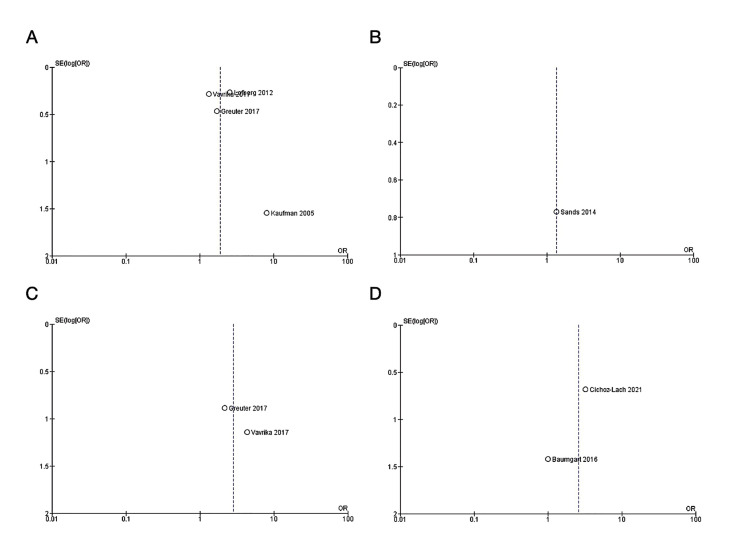
Funnel plot of comparison: A) Clinical evolution of AS under anti-TNF treatment in CD patients. B) Clinical evolution of AS under anti-integrin treatment in CD patients. C) Clinical evolution of AS under anti-TNF treatment in UC patients. D) Clinical evolution of AS under anti-integrin treatment in UC patients.

This could be due to the small number of studies included in each of the comparisons, given that no specific studies were found that evaluated the evolution of AS in these patients in isolation from the rest of the EIMs. However, a very similar pattern of behaviour was identified among the studies, with similar differences when looking at the effect of the specific comparisons, which makes the meta-analysis more solid (Fig. 4).

## Discussion

The results of the meta-analyses performed are in line with the published literature and with the systematic review carried out, showing a trend towards statistical significance in terms of the lower prevalence of AS in patients treated with anti-TNFs compared to those treated with anti-integrins (VDZ), however, the wide confidence intervals obtained when performing the analysis – probably related to the small number of studies available – and the impossibility of analysing all the studies included in this review due to the discrepancy between the variables analysed, makes it impossible to interpret these results and infer a solid conclusion from them. The minor influence on AS reduction after anti-integrin (VDZ) therapy may be due to altering the binding of leukocyte α4ß7 integrin to the mucosal addressing cell adhesion molecule-1 (MAdCAM-1) expressed in high endothelial venules of the gut, inhibiting the migration of leukocytes to the intestinal tract (20,39) and VDZ may redirect trafficking of α4ß7-expressing lymphocytes to other systems, predisposing these patients to develop EIMs with a parallel course to IBD (20,40). Other authors, such as Vavricka *et al*. (5) observed that AS was the third most responsive type of EIM to anti-TNF therapy (78.10%; n=25/32) and that the only predictor observed for a lower anti-TNF response was the presence of UC or IBD-U (OR=0.337; *p*=0.0139).

Despite the low overall prevalence of AS observed among the different studies, these Figures may be underestimated given that a high percentage of the included patients (up to 96%) were being concomitantly treated with corticosteroids (such as methylprednisone, prednisone or budesonide), immunomodulators (azathioprine, methotrexate, 6-mercaptopurine), anti-inflammatory drugs (sulfasalazine or 5-amino-salicylic acid [5-ASA]), antibiotics, and/or combinations of these. Nevertheless, Yi *et al*. (33) observed that patients treated with some of these therapies (intravenous injection of corticosteroids, immunosuppressants, and infliximab), before or recently, are the most susceptible to EIMs (*p*=0.012, 0.005, and 0.026, respectively). In contrast, patients not previously exposed to biological drugs are significantly less dependent on these drugs than those previously exposed (21).

It is important to consider that anti-TNF monoclonal antibodies are often used as first-line treatment in IBDs. However, in cases where an adequate response to treatment is not obtained, these drugs are replaced by anti-integrin therapies, such as VDZ. Therefore, a priori, we start from a "more unfavourable" situation to evaluate the effectiveness of anti-integrins in the reduction of EIMs such as AS. Furthermore, VDZ is the only IBD drug currently available that acts selectively in the intestinal tract, so its efficacy in the prevention of EIMs is presumed to be limited (18,20,21). On the other hand, patients on the second course of monoclonal therapy have a 28% increased risk of developing EIMs (adjusted IRR=1.28; 95% CI, 1.02–1.62) (8), however, as explained above, it is not possible to assess whether this risk is due to the unfavourable natural course of the disease or the therapy per se.

In approximately 38% of cases, AS appears before the diagnosis of IBD. The debut of EIMs before the onset of proper IBD clinical signs has been established as a positive prognostic factor for anti-TNF therapy (OR=9.70; 95% CI 1.04–90.04; *p*=0.046) (37). For this reason, dentists have a very important role in the prognosis of the disease and, in the presence of AS, underlying pathology such as IBD should be suspected. In this regard, intraoral examination of patients should be included as part of the physical examination performed by gastroenterologists, family doctors and/or paediatricians when IBD is suspected. The latter specialists are fundamental in the diagnosis of IBD in the paediatric population, a disease more prevalent in this group than in adults (8.50% vs. 5%, respectively; *p*=0.014).

Ulcers associated with AS in IBD are round, shallow, with a fibrous central membrane surrounded by an erythematous halo (24), usually located on the labial or oral mucosa, on the floor of the mouth and/or on the tongue (27). Differential diagnoses should be made with common aphthous ulcers, which also occur in patients with celiac sprue, HIV/AIDs, Behçet's disease, and Reiter's syndrome. Other entities with which to make the differential diagnosis are oral herpes simplex, Behçet's disease, and coxsackievirus infection. Although commonly confused with herpes simplex virus (HSV) lesions in their late stages, these lesions begin with vesicles that ulcerate, whereas aphthous ulcers do not have a vesicular phase. Coxsackievirus lesions also begin with vesicles, so, in case of doubt, Tzanck smear, antigen detection, culture, serology, or polymerase chain reaction can be performed to detect HSV. On the other hand, Behçet's disease is an idiopathic vasculitis that causes oral and genital ulcers, as well as ocular lesions such as uveitis and iritis (24). Typically, AS usually appear abruptly and coincides with a recurrence or exacerbation of IBD. Smaller aphthous ulcers (<10 mm) re-epithelialise without sequelae, while larger aphthous ulcers are deeper and often leave scars (27).

As this study has shown, treatment of the underlying pathology (IBD) usually results in remission of the ulcers (24,27), as AS usually develops in tandem with IBD, so its progression will follow that of the disease itself (8). However, the painful symptoms associated with AS can be alleviated by using topical anaesthetics, such as lidocaine 2% (24). Antiseptic rinses with chlorhexidine digluconate are recommended as it reduces pain by reducing bacterial colonisation of ulcers (27). Topical corticosteroids such as triamcinolone 0.10% paste, 1–3 times/day, or rinses with dexamethasone elixir 0.50 mg/5 mL, 1–3 times/day, can be administered to promote healing. Systemic corticosteroids should be reserved only in refractory cases or in severe or persistent ulcers. In addition, amlexanox 5%, a non-steroidal anti-inflammatory drug, can be used locally to promote healing and reduce associated pain (24).

- Strengths and limitations

This systematic review presents several strengths, such as the searching process of the studies, data extraction and risk analysis bias performed in duplicate, which determined a high overall quality of included studies. In addition, a large number of patients could be analysed, allowing for more solid conclusions to be drawn.

Nonetheless, the present study has limitations, such as heterogeneity between the different studies in terms of the disparity in the scales used to establish the severity/activity of IBD, variability in the previous/concomitant exposure of patients to different therapies that can improve the prevalence of AS, as well as the specification of the precise number of patients previously exposed or not to these therapies, and the absence in some studies of an adequate control group, which makes comparison difficult.

- Recommendations for further research

Future research should be designed homogeneously to be able to establish comparisons between them. Further study of patients diagnosed with IBD-U is also needed given the small sample size found in the literature and that they only assessed the effect of anti-TNF on the disease.

## Conclusions

Both anti-TNFs and anti-integrin (VDZ) monoclonal antibodies reduce the prevalence of AS in patients with IBD (CD and UC). However, the best results were obtained in patients treated with anti-TNFs. This association may be because VDZ is often used in patients who do not respond adequately to previous treatment with anti-TNFs and its specificity at the intestinal level. The effect of these therapies on the prevalence of AS in patients with IBD-U could not be determined.
